# History of the Ohio College of Dental Surgery

**Published:** 1859-03

**Authors:** 


### HISTORY OF THE OHIO COLLEGE OF DENTAL SURGERY.

See "Dental Register," Vol, VII, page 262. The name of James P. Hildreth, of Marietta, should be added as one of the Trustees.

In the spring of 1845, the Trustees met and organized, by appointing Rev. B. P. Aydelott, M. D., President, and Israel M. Dodge, M. D., Secretary.

Whereupon, they organized the school by creating the following Chairs, viz:

*Dental Anatomy* and *Physiology*—to which Jesse W. Cook, M. D., D. D. S., was appointed Professor.

*Dental Pathology* and *Therapeutics*—to which Melancthon Rogers, M. D., D. D. S., was appointed Professor.

*Practical Dentistry* and *Pharmacy*—to which James Taylor, M. D., D. D. S., was appointed Professor.

Jesse P. Judkins, M. D., was appointed Demonstrator of Anatomy, and Dr. James Taylor performed the duties of Demonstrator of Practical and Mechanical Dentistry.

The faculty, soon after their appointment, met and on ballot, Prof. Jesse W. Cook was elected Dean, who thereupon issued the first announcement, and the school was opened the first Monday of November, 1845.

The Faculty, on their organization, adopted the following Resolutions:

1. *Resolved*, That the term of each session of the School shall extend from the first Monday of November to the 20th of the following February.

2. *Rosolved*, That at the regular meeting at the close of each session, an election for Dean shall be held. And that the following, in relation to the requisitions for graduation—[See "American Journal of Dental Science," Vol. V, page 303.]

The opening address of the College was delivered by Rev. B. P. Aydelott, M. D., President. And the valedictory address to the graduating class by Prof. J. W. Cook.

February, 1846, the Faculty met and elected Prof. M. Rogers Dean; who, in June, issued the second annual announcement. Elijah Slack was by the Faculty appointed Lecturer on Chemistry; and the following resolution was passed by the Faculty:

*Resolved*, That no student shall be allowed the single ticket of any of the professors except those on Anatomy and Chemistry.

Valedictory to graduates, by Prof. M. Rogers.

February, 1847, Faculty met, and elected Prof. J. Taylor Dean; who, in due time, issued the third annual announcement.

October, 1847, Faculty met, and Prof. J. W. Cook tendered his resignation, which was by the Trustees accepted; and appointed to the Chair of Anatomy and Physiology, thus made vacant, J. F. Potter, M. D. The faculty appointed Dr. Wm. M. Hunter Demonstrator of Mechanical Dentistry. Jesse P. Judkins, M. D., resigned his place as Demonstrator of Anatomy, and Elijah Slack, M. D., continued to lecture on Chemistry. Valedictory to the graduates, by Prof. J. Taylor.

February, 1848, the faculty met, and Profs. J. F. Potter and M. Rogers resigned their respective Chairs; and no new election was held for Dean.

The Trustees met, during the summer, and having accepted the resignations of Profs. Potter and Rogers, appointed to the Chair of Dental Anatomy and Physiology, Prof. John T. Shotwell; and to the Chair of Dental Pathology and Therapeutics, George Mendenhall, M. D. The Faculty appointed A. M. Leslie, D. D. S., Demonstrator of Mechanical Dentristry. The Faculty appointed Charles H. Raymond, M. D., Lecturer on Chemistry. Valedictory to to the graduating class, by Prof. J. Taylor.

February, 1849, no election for Dean; but Dr. J. Taylor held over, and issued the annual announcement. Prof. Geo. Mendenhall delivered the valedictory to the graduating class.

February, 1850, no election for Dean was had. Prof. J. T. Shotwell resigned the Chair of Anatomy and Physiology. During the summer, the Trustees met; and having accepted the resignation of Prof. Shotwell, appointed to the Chair of Anatomy and Physiology, Thos. Wood, M. D.; and created a new Chair of Mechanical Dentistry, and appointed to the same, A. M. Leslie, D. D. S. The Faculty appointed Wm. H. King, D. D. S., Demonstrator of Mechanical Dentistry, and assistant Surgeon in the Infirmary. The Faculty passed the following resolution:

*Resolved*, That a committee of two from the medical and three from the dental profession be selected, annually, to examine, in connection with the Faculty, the candidates for graduation.

Whereupon, Charles Woodward, M. D., and Israel M. Dodge, M. D.. were selected from the medical, and Drs. N. Clute, W. E. Ide, and J. Allen, from the dental profession.

In December, Dr. A. M. Leslie resigned the Chair of Mechanical Dentistry, and Dr. J. Taylor, filled the same for the remainder of the session. Valedictory to the graduating class, by W. E. Ide, M. D.

February, 1851, no election for Dean,—Dr. J. Taylor held over. During the summer, the Trustees having accepted the resignation of Prof. A. M. Leslie, met and changed the Chair of Mechanical Dentistry to the Chair of Operative and Mechanical Dentistry, and appointed to the same, John Allen, D. D. S. They also changed the Chair of Practical Dentistry and Pharmacy to that of Principles and Practice of Dental Surgery, and appointed to the same, Dr. James Taylor.

The Faculty appointed G. L. Van Emen, D. D. S., Lecturer on Dental Chemistry, and Demonstrator of Operative and Mechanical Dentistry.

The following gentlemen were chosen a committee for the examination of the graduating class:

*Physicians.*—Profs. Jesse P. Judkins, M. D.; and L. M. Lawson, M. D.

*Dentists.*—Wm. M. Wright M. D., D. D. S.; H. R. Smith, D. D. S.; and E. Taylor, M. D., D. D. S.

Wm. M. Wright, M. D., delivered the valedictory to the graduating class.

February, 1852, Prof. J. Taylor was continued as Dean; who issued the annual announcement as usual. The examining committee, this year, was as follows:

*Dental.*—Dr. R. Somerby, Louisville, Ky.; Dr. E. Hale, St. Louis, Mo.; and Dr. A. L. Talbert, Dayton, Ohio.

*Medical.*—P. S. Conner, M. D., and P. G. Fore, M. D., Cincinnati, Ohio.

Valedictory to the graduates, by Prof. J Allen.

February, 1853, Prof. J. Taylor was continued as Dean. Prof. G. Mendenhall resigned the Chair of Pathology and Therapeutics, and his resignation was accepted by the Trustees, and J. B. Smith, M. D., was appointed to the same.

The Chair of Operative and Mechanical Dentistry was divided into two departments,—a Chair of Operative Dentisty, which was assigned to Prof. J. Allen; and a Chair of Mechanical Dentistry, to which was appointed H. R. Smith, D. D. S.

The Faculty appointed G. Watt, M. D., Lecturer on Chemistry.

Dr. G. L. Van Emon resigned his place as Demonstrator, and the duties were discharged by the Professor of Mechanical Dentistry. The examining committee were—

*Dental.*—A. L. Talbert, D. D. S., Lexington, Ky.; W. H. Goddard, D. D. S., Louisville, Ky.; and H. E. Peebles, D. D. S., Lexington, Mo.

*Medical.*—Prof. G. Mendenhall, and Israel M. Dodge, M. D., Cincinnati, Ohio.

Valedictory to the graduating class, by Prof. H. R. Snith.

February, 1854. Prof. J. Taylor was continued Dean of Faculty. Prof. J. Allen resigned the Chair of Operative Dentistry; the resignation was accepted by the Trustees, and Jonathan Taft, D. D. S., was appointed to the same.

The Faculty appointed George M. Kellogg, M. D., Lecturer on Chemistry. The examining committee were—

*Dental.*—J. P. Ulrey, D. D. S., Rising Sun, Ind.; J. G. Hamill, D. D. S., Georgetown, Ky.; and C. H. Quinlan, Chicago, Ill.

*Medical.*—Profs. L. M. Lawson, and John Davis, Cincinnati, Ohio.

*Janitor*—Mr. Joseph Edwards.

Address to the graduating class, by Prof. J. Taylor.

February, 1855, Faculty met and elected Dr. J. Taylor, Dean.

Prof. T. Wood resigned the Chair of Anatomy and Physiology, which was accepted by the Trustees, and Prof. C. B. Chapman was appointed to the same.

The Trustees changed the Chair of Principles and Practice of Dental Surgery, to that of Institutes of Dental Science, and assigned to the same, Dr. J. Taylor. They also created a Chair of Chemistry and Metallurgy, and appointed to the same, Dr. George Watt, D. D. S. The examining committee were—

*Dental.*—Drs. J. P. Ulrey, T. R. Willard, and G. W. Keely.

*Medical.*—Profs. W. H. Mussey, and C. M. Langdon.

Address to the graduating class, by Prof. J. Taft.

At the close of the session of 1855–56, Dr. Taylor declined being a candidate for Dean. On balloting, it was found that Geo. Watt was duly elected Dean of the Faculty, for the session of 1856–57. The annual announcement was issued in July, under the same Faculty as that of last year. Dr. H. A. Smith of Oxford, Ohio, was appointed Demonstrator of Operative and Mechanical Dentistry.

The session opened, as usual, on the first Monday of November, and closed the 25th of February ensuing. The Commencement exercises were conducted in the lecture-room of the College, on the evening of the said 25th of February,—Rev. Dr. Aydelott, president of the Board of Trustees, conferring the Degrees, and presenting each graduate, as usual, with a copy of the Holy Bible. He also delivered an address to the graduates, on "The Professional Studies of the Dental Pupil;" and Dr. Watt delivered the "valedictory," on behalf of the Faculty.

At the close of this session, Prof. H. R. Smith resigned the Chair of Mechanical Dentistry. The College Association recommended Dr. Joseph Richardson, of Cincinnati, to the Board of Trustees, as a suitable person to fill the vacancy; and he was, in due time, elected to said Chair, by the Board. Dr. G. Watt was re-elected Dean of the Faculty for 1857–58.

The annual announcement of the session of 1857–58 was issued in July. The session began the first Monday of November, and closed the 17th of February following.

The Degrees, as usual, were conferred by President Aydelott; who addressed the candidates on "The Ethics of the Dental Profession." The "valedictory" was delivered by Prof. Chapman. Dr. J. Taft was elected Dean, for session of 1858–59.

The fourteenth annual announcement was issued early in July. The Faculty remained as during last session. Dr. H. A. Smith was continued as Demonstrator.

The session of 1858–59 opened with a preliminary course on the 18th of October; and the regular course, on the first Monday of November. The class numbered twenty-three. There were nine graduates. The commencement exercises took place on the 23d of February. Dr. B. P. Aydelott, President, conferred the Degrees, and delivered an address.—The address to the graduates was delivered by Dr. W. W. Allport, of Chicago.

LIST OF STOCKHOLDERS OF THE OHIO COLLEGE OF DENTAL SURGERY.

| | |
|---|---|
| James Taylor, Cincinnati, Ohio, | C. W. Spaulding, St. Louis, Mo., |
| Chas. Bonsall, " " | H. E. Peebles, " " |
| A. Berry, Raymond, Miss., | H. J. B. McKellops, " " |
| G. L. Vanemon, Cincinnati, Ohio, | J. S. Clark, New Orleans, La., |
| W. C. Duncan, " " | James Knapp, " " |
| Edward Taylor, " " | Chas. E. Keels, " " |
| Thos. Wood, " " | W. S. Chandler, Port Gibson, Miss. |
| Geo. Mendenhall, " " | David Dougherty, Bardstown, Ky. |
| J. B. Smith, " " | M. N. Manlove, Logansport, Ind. |
| J. M. Brown, " " | J. P. Ulrey, Rising Sun, Ind. |
| Samuel Wardle, " " | J. W. Baxter, Warsaw, Ky. |
| John Allen, New York, N. Y., | E. Bray, Evansville, Ind. |
| Wm. M. Wright, Pittsburg, Pa., | E. A. Herman, Nashville, Tenn. |
| H. R. Smith, Cincinnati, Ohio, | Moses De Camp, Mansfield, Ohio, |
| Jon. Taft, " " | Geo. W. Keely, Oxford " |
| A. S. Talbert, Lexington, Ky. | Jones, White & McCurdy, Phila., |

J. B. Dunlevy, Pittsburg, Pa.
Wm. R. Webster, Richmond, Ind.
George Watt, Xenia, Ohio,
Joseph Richardson, Cincinnati, O.
H. A. Smith, Oxford, Ohio,
Jas. S. King, Pittsburg, Pa.
G. J. Fredricks, New Orleans, La.
A. J. Reeve, Mt. Vernon, Ohio,
Edgar Taylor, Palmyra, Mo.
Samuel Griffith, Louisville, Ky.
J. B. Miner, Milwaukee, Wis.
John T. Toland, Cincinnati, Ohio,
C. B. Chapman, Madison, Wis.
W. W. Allport, Chicago, Ill.
David W. Perkins, Milwaukee, Wis.
I. B. Branch, Galena, Ill.
B. B. Ward, Mobile, Ala.
J. M. Lewis, Marion, Ill.
Eli Collins, Connersville, Ind.

At the annual meeting of the Association, Feb. 1855, each member was voted a share of stock.

James Taylor,

Thos. Wood,

J. B. Smith,

Jon. Taft,

H. R. Smith,

List of those who have been elected members of the Ohio Dental College Association and are not stockholders.

J. F. Johnston, Indianapolis, Indiana,
J. L. Milton, Lexington, Mississippi,
A. L. Dwyer, Greenfield, Ohio.

### DENTAL QUACKS, NO. 3—CONTINUED.

BY H. R. SMITH.

